# Current Evidence on the Impact of Diet, Food, and Supplement Intake on Breast Cancer Health Outcomes in Patients Undergoing Endocrine Therapy

**DOI:** 10.3390/nu17030456

**Published:** 2025-01-26

**Authors:** Milena Žuža Praštalo, Biljana Pokimica, Aleksandra Arsić, Jasminka Z. Ilich, Vesna Vučić

**Affiliations:** 1 Group for Nutritional Biochemistry and Dietology, Institute for Medical Research, National Institute of Republic of Serbia, University of Belgrade, 11000 Belgrade, Serbia; milena.zuza@imi.bg.ac.rs (M.Ž.P.); biljana.pokimica@imi.bg.ac.rs (B.P.); aleksandra.arsic@imi.bg.ac.rs (A.A.); vesna.vucic@imi.bg.ac.rs (V.V.); 2 Centre of Research Excellence in Nutrition and Metabolism, Institute for Medical Research, National Institute of Republic of Serbia, University of Belgrade, 11000 Belgrade, Serbia; 3 Institute for Successful Longevity, Florida State University, Tallahassee, FL 32306, USA

**Keywords:** breast cancer, estrogen/progesterone-positive breast cancer, endocrine adjuvant therapy, aromatase inhibitors, tamoxifen, bone and body composition, cardiovascular risk factors, inflammation, quality of life

## Abstract

Background/Objectives: The most common type of breast cancer (BRC) in women is estrogen/progesterone receptor positive. First-line treatment includes endocrine therapy, either with aromatase inhibitors or tamoxifen to reduce estrogen levels. Among the side effects produced by this treatment, aromatase inhibitor-induced arthralgia is the most common, affecting the patients’ overall health and quality of life (QoL). The objectives here were to evaluate interventions examining the impact of modified diets, supplements, and/or some food components on health outcomes in BRC patients undergoing endocrine therapy. Methods: The literature search was performed in PubMed, Scopus, and Web of Science from June 2024, as well as manually, through the end of November 2024. The search was limited to studies of women diagnosed with estrogen/progesterone-receptor-positive BRC with selected articles reporting interventions with diet, food, or supplement intake and examining the relevant health outcomes. Studies not focusing on BRC patients undergoing endocrine therapy or not including specific health outcomes were excluded. Results: The search uncovered 1028 studies; after the removal of duplicates, abstracts, and irrelevant studies, 53 were closely examined, with 26 evaluated and presented here. The outcomes were changes in bone and body composition, cardiovascular disease risks, inflammation, and QoL. Conclusions: The examined evidence suggests that adherence to dietary patterns such as the Mediterranean or a low-fat diet, and a higher intake of fruits and vegetables were beneficial for various outcomes. Additionally, supplementation with some foods/components (dried plum, red clover) contributed to improving/maintaining bone and body composition, especially in overweight/obese patients. Supplementation with vitamin D or omega-3 improved lipid and angiogenic parameters and QoL. Although these results are promising, the effects of each supplement/food cannot be summarized due to the diverse nature of study designs, patients, and supplement dosages. Further studies are needed to explore the effects of specific nutritional interventions (including the newest, like fasting-mimicking diets and whole-grain cereal diets) on various health outcomes in BRC survivors during endocrine therapy, and to derive universal recommendations.

## 1. Introduction

Breast cancer (BRC) is a prevalent disease affecting women around the globe. It is the most common cancer and the leading cause of cancer-related deaths among women. In 2020, approximately 2.3 million new cases of BRC were diagnosed worldwide, accounting for nearly a quarter of all cancer cases in females [1,2,3].

The non-modifiable risk factors for BRC include female sex, older age, family history of BRC, certain genetic mutations, dense breast tissue, and previous radiation exposure. Women at higher risk for developing BRC are those with early menarche (before age 12), late menopause (after age 55), those not having children, or having a first pregnancy after age 30 [4,5]. The modifiable risk factors include the use of certain medications, high body mass index (BMI), alcohol consumption, smoking, exposure to artificial light or environmental pollutants, as well as physical inactivity, and a nutrient-poor diet, including the consumption of highly processed foods and a low intake of vitamins and minerals [4]. As Western dietary patterns, characterized by the increased consumption of processed, sugary, and high-fat foods, continue to spread globally, the incidence of BRC may rise in both developed and developing countries. Therefore, addressing these modifiable factors is essential in the search for preventive or new therapeutic strategies for BRC [4,5].

About 75% of breast tumors express either the estrogen or progesterone receptor, classifying them as hormone-receptor-positive (HR+) tumors. Adjuvant endocrine therapy is a fundamental component of treatment for HR+ early BRC, alongside chemotherapy and radiation therapy [6]. Since estrogen significantly contributes to the proliferation of cancer cells in patients with estrogen receptor breast cancer, blocking it from binding to its receptors became an effective treatment. The most common endocrine treatments include tamoxifen (TAM) and/or aromatase inhibitors (AIs). TAM blocks estrogen’s ability to attach to breast cancer cells, while AI hinders the aromatase enzyme that converts other androgens to estrogen. Both treatments result in a reduction in estrogen levels and slow down the growth of breast cancer cells [7,8,9,10]. In early premenopausal HR and breast cancer patients, AIs such as anastrozole, exemestane, or letrozole have shown lower recurrence rates compared to tamoxifen [11].

Although the side effects from endocrine treatments are less severe compared to chemo or radiation therapy, there are quite a few side effects that could affect overall health and quality of life, including a resurgence of menopausal symptoms in women who are postmenopausal, as well as the development of metabolic syndrome, increased pro-inflammatory C-reactive protein, and non-alcoholic steatohepatitis [12]. The evidence shows the effectiveness of AI treatment in enabling cancer-free survival for breast cancer patients; however, its use can be limited because of debilitating but otherwise modifiable side effects. For example, nearly 50% of patients on AIs experience arthralgia, including joint pain, stiffness, and muscle pain (arthralgia and myalgia), often as part of a broader condition called AI-induced musculoskeletal syndrome, which also includes bone loss and increased fracture risk, all primarily due to estrogen deficiency. Moreover, AI treatment is associated with other side effects, such as cognitive dysfunction, anxiety, depression, sleep disturbances, and fatigue. It can also lead to changes in lipid profiles and elevated cardiovascular risk, as well as changes in the urogenital system, causing sexual dysfunction and frequent urinary tract infections [13,14]. Regarding TAM therapy, in addition to arthralgia, its long-term use can lead to venous thromboembolism, secondary cancers (e.g., uterine), central nervous system damage, and bone growth abnormalities [15]. Consequently, many women undergoing endocrine therapy describe it as a paradox: “a life-saving treatment that also contributes to premature aging”.

Overall, while both types of treatment provide significant benefits in preventing cancer recurrence, their side effects can negatively impact a patient’s quality of life (QoL) and contribute to nonadherence to treatment and therapy discontinuation. Therefore, it is crucial to address and minimize the negative effects of both therapies to improve overall health and QoL for breast cancer patients [13]. Increasing evidence from research on various dietary patterns, macro- and micronutrients, and bioactive compounds points to the significance of nutrition interventions in improving treatment outcomes for breast cancer patients and survivors. For example, certain dietary factors like excess carbohydrates (especially simple sugars), saturated fats, and red meat may increase breast cancer risk. Conversely, nutrients that contain fiber, omega-3 fatty acids, some vitamins, minerals, and phytochemicals found in fruits and vegetables, as well as in whole-grain cereals, may help reduce oxidative stress and chronic inflammation, both known to contribute to breast cancer development and progression [4,5,16,17,18,19].

Both scientific and clinical communities and patient advocacy groups emphasize the importance of lifestyle changes post diagnosis, particularly regarding diet. In this context, a growing concern is the increasing number of breast cancer survivors (BRCS) who are overweight or obese. BRCS with overweight (BMI 25–29.9) or obesity (BMI > 30) tend to have worse outcomes compared to those with a normal BMI (<25), including higher recurrence rates, poorer treatment responses, and an increased risk of secondary cancers and metastases. The results from a recent meta-analysis revealed that women with obesity face a 33% higher risk of breast cancer mortality, and a 41% higher risk of overall mortality compared to those with a normal weight [17]. Research suggests that BRCS who adopt a healthier lifestyle—such as increasing physical activity and improving diet and overall dietary habits—may experience better QoL and potentially improve their overall health, thereby prolonging life and reducing the healthcare burden [17,18,19]. In line with this, a very comprehensive and excellent review on the nutritional management of many oncological symptoms in various cancers, along with a multidisciplinary roadmap incorporating some customized recipes and recommendations, have been published recently [18]. Similarly, a comprehensive review of the potential of whole-grain cereal diets in preventing and/or curtailing breast cancer was published earlier [19].

The objectives of this review were to systematically synthesize the available evidence on the impact of the modified diet, supplements, and some food components on breast cancer outcomes in patients undergoing treatment with AIs or TAM. By reviewing and evaluating interventional studies, we identified key findings that could inform clinical practice and guide future research. Our analysis also highlighted the potential role of nutrition and supplementation in optimizing outcomes during first-line cancer treatment and later, offering insights into how these strategies could complement conventional cancer therapies, with a focus on body composition, cardiovascular disease risk parameters, inflammation, and quality of life. These findings could help shape future clinical guidelines and highlight areas where further investigation is needed to enhance patient care and survivorship, possibly contributing to universal treatments in alleviating endocrine treatment side effects.

## 2. Materials and Methods

### 2.1. Search Strategy

The literature search was performed from June 2024 across three scientific databases: PubMed, Scopus, and Web of Science, from the article’s inception to the end of September 2024. A combination of the following MeSH keywords was used: breast cancer AND food OR diet OR supplement AND aromatase inhibitors OR tamoxifen. The bibliography section of the included studies was also manually screened to find additional relevant studies, as well as the applicable articles that were in print or published after the search was completed, extending to the end of November 2024.

### 2.2. Selection Criteria

The studies included in this review were intervention studies that reported the effects of a whole or modified diet, food components, or supplement intake (including minerals and vitamins) on the health outcomes of adult female BRC patients who took AIs or TAM as a treatment. Therefore, the relevance of the studies was determined based on whether the study focused on the effects of diet, food, or supplement intake specifically in BRC patients undergoing endocrine therapy, and whether the health outcomes assessed aligned with the review’s objectives (namely, for body composition, cardiovascular disease, inflammation, and QoL). Only studies published in the English language were used, and no time limitation was applied. The exclusion criteria were as follows: case reports, review articles, book chapters, conference abstracts or abstracts only, letters, and articles with unusable information. To avoid any potential bias, the screening was performed independently by two authors (M.Ž.P. and B.P.), who screened the titles and abstracts and evaluated the full-text articles for relevance. Based on the predefined inclusion and exclusion criteria, the authors subsequently selected potentially eligible articles, with a 10% double checking. Two authors (M.Ž.P. and B.P.) independently extracted the data from the included articles.

## 3. Results

**Figure 1:**
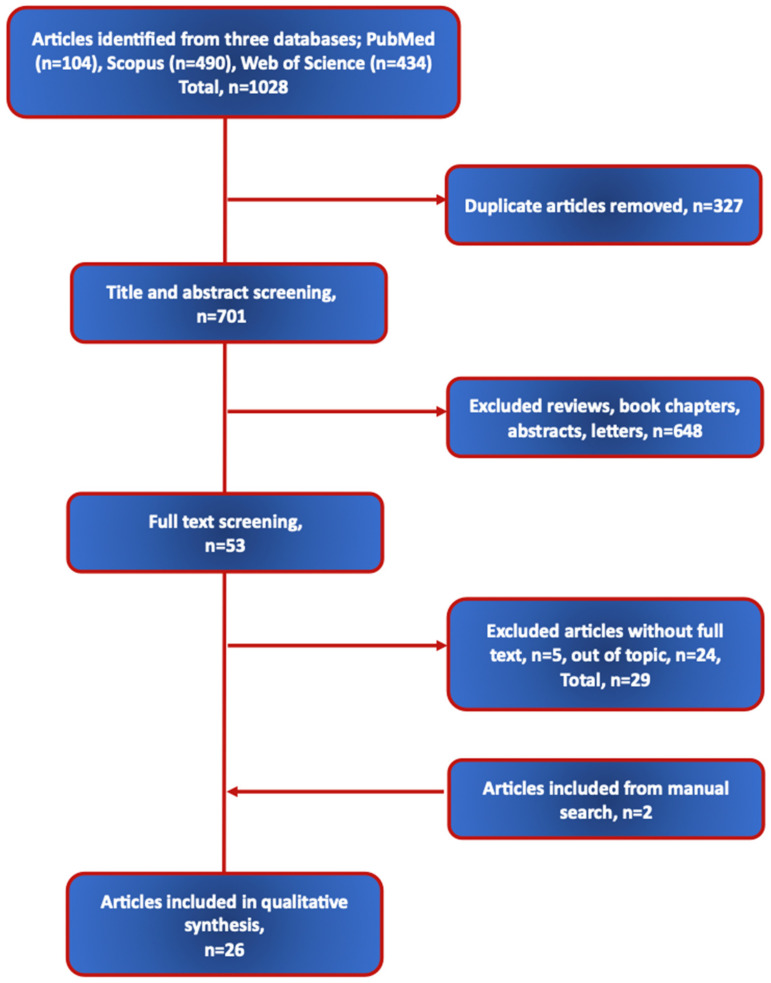
Flowchart of the search process.

The selection process is presented in Figure 1, with 26 eligible articles evaluated and discussed. Each study’s characteristics and main findings are summarized in Table 1, Table 2, Table 3, Table 4 and Table 5, designed based on the type of intervention to include the following: modified diets; omega-3 fatty acids; vitamin/mineral supplements; plant/fungi food and supplements; and coenzyme Q_10_.

### General Characteristics of the Studies

Most of the studies were conducted in the USA (n = 9), followed by India (n = 4), Iran, Spain and Italy, each (n = 2), Greece, Iraq, Mexico, South Korea, The Netherlands, Taiwan, and the United Kingdom, each (n = 1) (Table 1, Table 2, Table 3, Table 4 and Table 5).

The intervention included specialized diets [20,21,22], omega-3 fatty acid supplements [23,24,25,26], and vitamins and minerals [27,28,29,30,31,32,33]. Also, some of the included studies investigated plant-based food (dried plum) [34] and soy milk (isoflavones) [35], as well as supplements, mostly rich in polyphenols, such as resveratrol, curcuminoids, lignans [36], red clover isoflavones [36,37], soy isoflavones [38], and green tea epigallocatechin gallate [39], as well as a combination of compounds with anti-inflammatory potential (α-lipoic acid, Boswellia serrata, methylsulfonylmethane, and bromelain) [40]. Other studies investigated supplements based on immunomodulatory protein from the medicinal fungi Ganoderma [41] and coenzyme Q_10_ (CoQ_10_) [42,43,44,45].

Only three studies included intervention with physical activity/exercise [21,22,34], namely resistance and aerobic activity [21] or strength training and resistance [34], or the incorporation of the physical activity guidelines [22]. Since in two of these studies [22,34] the effects of physical activity and diet were assessed together, and the third study included overall lifestyle modification (diet and physical activity), we decided to include all three studies in this review.

All included studies are described in Table 1, Table 2, Table 3, Table 4 and Table 5, providing comprehensive information on the kind/design of research, number and characteristics of subjects, previous or current therapy, duration, food components, the whole diet or supplements used, the evaluated parameters, and the main results/significant changes. However, only the outcome parameters analyzed in two or more studies are discussed below.

**Table 1:** Characteristics and results of studies with modified type of diet.

Author; Year	Country	Participants: Mean/Median Age; BRC Stage; Use of AI/TAM; Menopausal Status	Groups: n of Analyzed Samples; Duration of the Study	Evaluated Parameters	Significant Changes
Modified Diets					
Brown et al., (2021) [21]	Pennsylvania, USA	Mean, 59.4 y; Stage I-IIIA BRC; Completed chemotherapy, radiotherapy, and targeted therapy for ≥6 months before baseline data collection; AI/TAM	1. Exercise plus diet (E and D) (n = 87) 2. Exercise only (E) (n = 87) 3. Diet only (D) (n = 87) 4. Control (C) (n = 87) D: Weeks 0–20: meal replacement program (7 servings of fruits and vegetables daily) Weeks 21–24: behavioral modification regarding shopping and preparation of food Weeks 24–52: behavioral modification regarding problem solving and prevention of relapse E: Weeks 0–6: exercise (resistance and aerobic activity) instructions Weeks 7–52: diet instructions	**Physical and mental health** (SF-36)	**↑Physical health summary score** E + D vs. C (*p* < 0.05) **↑Physical functioning** E + D vs. C (*p* < 0.05) **↑Role–physical** E + D vs. C (*p* < 0.05) **↑Vitality** E + D vs. C (*p* < 0.05) D vs. C (*p* < 0.05)
Murillo Ortiz et al., (2017) [20]	Mexico	Mean; Diet, 50.5 y; Control, 52.3 y; TAM; Postmenopausal	1. Diet (D) (n = 50) 12% fat, 68% carbohydrates, and 20% protein; Written instructions 2. Control (C) (n = 50) 30% fat, 50% carbohydrates, and 20% protein; Written instructions Duration: 6 months	**Body composition** (BMI, WC, muscle mass) **Blood parameters** (estradiol, adiponectin, glucose, IGF-1)	**Body composition** **↓BMI** D vs. C (*p* = 0.04) D end vs. baseline (*p* = 0.01) **↓WC** D end vs. baseline (*p* = 0.0001) **Blood parameters** **↓Estradiol** D end vs. baseline (*p* = 0.003) **↓Adiponectin** D end vs. baseline (*p* = 0.004) **↓Glucose** D end vs. baseline (*p* = 0.0001)
Papandreou et al., (2021) [22]	Greece	Mean, 49.7 y; Stage I-IIIA BRC; AI/TAM; Postmenopausal	1. Diet (D) (n = 22) personalized daily dietary plan based on the Mediterranean diet and physical activity guidelines, generated by CDSS; Written instructions 2. Control (C) (n = 22) general lifestyle advice Duration: 3 months	**Body composition** (BMI, BW, %BFM, WC) **Physical activity levels (metabolic equivalent-min/week)** **Blood parameters** (glucose, TAG, total cholesterol, LDL, HDL) **Oxidative stress** (MDA levels, plasma vitamin C) **Quality of life** (EORTC-QLQ-C30, EORTC QLQ-BR23, HADS) **Adherence to Mediterranean diet**	**Body composition** **↓BW** D end vs. baseline (*p* < 0.001) D vs. C (*p* < 0.001) **↓BMI** D end vs. baseline (*p* < 0.001) D vs. C (*p* < 0.001) **↓%BFM** D end vs. baseline (*p* < 0.001) D vs. C (p < 0.001) **↓WC** D end vs. baseline (*p* < 0.001) D vs. C (*p* < 0.001) ↑**Physical activity levels** D end vs. baseline (*p* = 0.001) D vs. C (*p* = 0.001) **Blood parameters** **↑Glucose** C vs. D (*p* = 0.043) C end vs. baseline (*p* = 0.046) **↑TAG** C vs. D (*p* = 0.008) C end vs. baseline (*p* = 0.016) **↑Total cholesterol** C end vs. baseline (*p* = 0.045) **↑LDL** C end vs. baseline (*p* = 0.037) **↑HDL** D end vs. baseline (*p* = 0.034) **Oxidative stress** **↑MDA levels** C vs. D (*p* = 0.007) C end vs. baseline (*p* = 0.017) **↑Plasma vitamin C** D baseline vs. end (*p* < 0.001) D vs. C (*p* = 0.021) **Quality of life** **↑EORTC-QLQ-C30 global health-quality of life scale** D end vs. baseline (*p* = 0.035) **↑EORTC-QLQ-C30 role functioning subscale** D end vs. baseline (*p* = 0.047) **↑EORTC-QLQ-C30 emotional functioning subscale** D end vs. baseline (*p* = 0.037) **↓HADS depression scale** **D end vs. baseline** (*p* = 0.022) **↓HADS anxiety scale** **D end vs. baseline** (*p* = 0.022) **Adherence to Mediterranean diet** **↑MedDiet score** D end vs. baseline (*p* < 0.001) D vs. C (*p* = 0.002)

**Abbreviations:** AIs: aromatase inhibitors; BFM: body fat mass; BMI: body mass index; BRC: breast cancer; BW: body weight; CDSS: clinical decision support systems; EORTC QLQ-BR23: European organization for research and treatment of cancer quality of life questionnaire the breast cancer supplementary module; EORTC QLQ-C30: European organization for research and treatment of cancer quality of life questionnaire core 30; HADS: hospital anxiety and depression scale; HDL: high-density lipoprotein; IGF-1: insulin-like growth factor 1; LDL: low-density lipoprotein; MDA: malondialdehyde; MedDiet score: Mediterannean diet score; (SF)-36: medical outcome survey short form; TAG: triglycerides; TAM: tamoxifen; WC: waist circumference; ↑: an increase or improvement in the associated value or measurement; and ↓: a decrease or deterioration in the associated value or measurement.

**Table 2:** Characteristics and results of studies with omega-3 fatty acids.

Author; Year	Country	Participants: Mean/Median Age; BRC Stage; Use of AI/TAM; Menopausal Status	Groups: n of Analyzed Samples; Duration of the Study	Evaluated Parameters	Significant Changes
Omega-3 Fatty Acids					
Hershman et al., (2015) [23]	Multicenter, USA	Mean, 59.2; Stage I-III BRC; AI for at least 90 days before baseline; Postmenopausal	1. Omega-3 (560 mg of EPA and DHA) (n = 102) 2. Placebo (soybean and corn oil) (n = 107) Duration: 24 weeks	**Serum parameters** (TAG, total cholesterol, HDL, LDL) **Serum markers of inflammation** (CRP) **Quality of life** (FACT-ES) **Pain/stiffness** (BPI worst pain score, BPI pain interference)	**Blood parameters** **↓TG** Omega-3 vs. placebo (*p* = 0.01) **Pain/stiffness outcomes** **↓BPI scores** Omega-3 end vs. baseline (*p* < 0.001) Placebo end vs. baseline (*p* < 0.001) **BPI scores** Omega-3 vs. placebo (*p* > 0.05)
Hutchins-Wiese et al., (2014) [24]	Connecticut, USA	Mean; Omega-3, 60.9 y; Placebo, 63.6 y; AI for at least 6 months before baseline	1. Omega-3 (n = 17): 4 g EPA and DHA (2520 mg EPA, 1680 mg DHA) and calcium carbonate (1000 mg/day) and cholecalciferol (800 IU/day) 2. Placebo (n = 17): safflower oil (9% linoleic acid, 83% oleic acid) and calcium carbonate (1000 mg/day) and cholecalciferol (800 IU/day) Duration: 3 months	**Bone markers** (sCTX, DPD, P1NP, BAP, PTH) **Serum markers of inflammation** (IL-6, IL-1 β, hsCRP, migratory inhibitory factor, TNFα) **Serum fatty acid concentrations**	**Bone markers** ↓**sCTX levels** Omega-3 (responders) vs. placebo (*p* = 0.038) Omega-3 (responders) end vs. baseline (*p* < 0.05) **↓DPD** Omega-3 vs. placebo (*p* = 0.043) Omega-3 end vs. baseline (*p* < 0.05) **↓P1NP** Omega-3 end vs. baseline (*p* < 0.05) **↓BAP** Omega-3 end vs. baseline (*p* < 0.05) **Blood markers of inflammation** ↑hsCRP Omega-3 vs. placebo (*p* = 0.022) Omega-3 end vs. baseline (*p* = 0.027) **Fatty acid status in blood** **↑Total and LC omega-3 PUFA** Omega-3 end vs. baseline Omega-3 vs. placebo (*p* < 0.001) **↓Total and LC omega-6 PUFA levels** Omega-3 end vs. baseline Omega-3 vs. placebo (*p* < 0.001)
Martínez et al., (2019) [25]	Spain	Median, 57.3 y; Stage 0-IIIA BRC; AI/TAM for at least 3 months before baseline; Postmenopausal	1. Omega-3 (460 mg) and hydroxytyrosol (12.5 mg) and curcumin (47.5 mg curcuminoids) (n = 45) Duration: 30 days	**Blood parameters** (TAG, LDL, HDL) **Blood markers of inflammation** (CRP, IL-6, SAA, IFNγ, TNFα, IL-10, IL-15, TGFβ, IGF-1) **Pain outcomes** (BPI-SF)	**Blood parameters** ↓TG Omega-3 end vs. baseline (*p* = 0.011) **Blood markers of inflammation** **↓CRP** Omega-3 end vs. baseline (*p* = 0.014) **↓IFNγ** Omega-3 end vs. baseline (*p* = 0.056) **Musculoskeletal pain** **↓BPI worst pain score** Omega-3 end vs. baseline (*p* = 0.011) **↓Pain severity index score** Omega-3 end vs. baseline (*p* = 0.008)
Lustberg et al., (2018) [26]	Ohio, USA	Mean, 59.5 y; Stage I-III BRC; AI for less than 21 days before enrollment; Postmenopausal	1. Omega-3 (4.3 g) (n = 22) 2. Placebo (n = 22) Duration: 24 weeks	**Blood markers of inflammation** (IL-6, TNFR-2, IL-17) **Fatty acids status in red blood cells** **Quality of life** (FACT-ES) **Pain outcomes** (BPI-SF)	**Fatty acids status** **↑LC omega-3** Omega-3 end vs. baseline (*p* < 0.001) **↓Total omega-6 PUFA** Omega-3 end vs. baseline (*p* < 0.001) **Quality of life** **↓FACT-ES scores** Placebo 12 weeks vs. baseline (*p* = 0.04) Placebo vs. omega-3 12 weeks (*p* = 0.06)

**Abbreviations:** AIs: aromatase inhibitors; BAP: bone specific alkaline phosphatase; BPI-SF: brief pain inventory–short form; BRC: breast cancer; CRP: C-reactive protein; DHA: docosahexaenoic acid; DPD: deoxypyridinoline; EPA: eicosapentaenoic acid; FACT-ES: functional assessment of cancer therapy–endocrine symptoms; HDL: high-density lipoprotein; hsCRP: high-sensitivity C-reactive protein IFNγ: interferone gamma; IGF-1: insulin-like growth factor 1; IL-1: interleukin-1; IL-10: interleukin-10; IL-15: interleukin-15; IL-17: interleukin-17; IL-6: interleukin-6; LC: long chain; LDL: low-density lipoprotein; P1NP: procollagen type 1 N-terminal propeptide; PTH: parathyroid hormone; SAA: serum amyloid A; sCTX: serum C-terminal telopeptide; TG: triglycerides; TAM: tamoxifen; TGFβ: transforming growth factor beta; TNFα: tumor necrosis factor alpha; TNFR-2: tumor necrosis factor receptor 2; ↑: an increase or improvement in the associated value or measurement; and ↓: a decrease or deterioration in the associated value or measurement.

**Table 3:** Characteristics and results of studies with supplemental minerals and vitamins.

Author; Year	Country	Participants: Mean/Median Age; BRC Stage; Use of AI/TAM; Menopausal Status	Groups: n of Analyzed Samples; Duration of the Study	Evaluated Parameters	Significant Changes
Minerals and Vitamins					
Campbell et al., (2018) [31]	Texas, USA	Stage I-III BRC; AI; Postmenopausal	1.Vitamin B_12_ (2500 mcg) (n = 41) Duration: 3 months	**Quality of life** (FACT-ES) **Pain outcomes** (BPI-SF) **Blood markers of inflammation** (CRP, HCys, MMA) **Serum B12 levels**	**Quality of life** **↑ Physical well-being** Vitamin B_12_ end vs. baseline (*p* < 0.0001) **↑Endocrine symptom subscale** Vitamin B_12_ end vs. baseline (*p* < 0.0001) **Pain outcomes** **↓Worst pain score** Vitamin B_12_ end vs. baseline (*p* = 0.0003) **↓Average pain scores** Vitamin B_12_ end vs. baseline (*p* < 0.0001) **Blood markers of inflammation** **↓HCys** Vitamin B_12_ end vs. baseline (*p* < 0.0001) **↓MMA** Vitamin B_12_ end vs. baseline (*p* < 0.0001) ↑**Serum B12 levels** Vitamin B_12_ end vs. baseline (*p* < 0.0001)
Khan et al., (2017) [29]	Kansas, USA	Median, 61 y; Stage I-III BRC; Scheduled to start treatment with AI; Postmenopausal	1. Vitamin D (n = 70) 30,000 IU oral vitamin D3/week and calcium 1200 mg and 600 IU of vitamin D 2. Placebo (n = 77) matched capsules and calcium 1200 mg and 600 IU of vitamin D Duration: 24 weeks	**Serum vitamin D** **Quality of life** (BFI, FACT-B, MENQOL) **Pain outcomes** (HAQ-II, CPIS, BPI) **Grip strength**	↑**Serum vitamin D levels** Vitamin D vs. placebo (*p* < 0.001) Vitamin D end vs. baseline (*p* < 0.001) **Pain outcomes** ↑Worsening of AIMSS Placebo vs. vitamin D (only post hoc, *p* = 0.024)
Niravath et al., (2019) [32]	Texas and Washington, USA	Median, 64 y; Stage I-III BRC; Scheduled to start treatment with AI; Postmenopausal	1. Standard-dose vitamin D3 (800 IU daily) and calcium carbonate 600 mg (n = 47) 2. High-dose vitamin D3 (50,000 IU weekly for 12 weeks, followed by 2000 IU daily for 40 weeks) and calcium carbonate 600 mg (n = 46) Duration: 52 weeks	**Serum vitamin D** **Pain outcomes** (HAQ-II scores) **Grip strength**	↑**Serum vitamin D levels** High-dose vitamin D vs. standard-dose vitamin D at 12 weeks (*p* < 0.0001) High-dose vitamin D 12 weeks vs. baseline (*p* < 0.0001) **Pain outcomes** **↑AI-induced arthralgia** developed in both groups in ~50% of patients
Park et al., (2011) [28]	Virginia, USA	Mean, 53.5 y; TAM/AI; Postmenopausal	1. Magnesium (n = 25) (400 mg once a day first 2 weeks, those whose symptoms did not improve used 2× day next 2 weeks) Duration: 4 weeks	**Quality of life** **(menopausal symptoms)**	**Quality of life** **Menopausal symptoms** **↓Hot flash frequency** Magnesium end vs. baseline (*p* = 0.02) **↓Hot flashes score** Magnesium end vs. baseline (*p* = 0.04) **↓Level of fatigue** Magnesium end vs. baseline (*p* = 0.03) **↓Abnormal sweating** Magnesium end vs. baseline (*p* = 0.0004) **↓Perceived distress level due to hot flashes** Magnesium end vs. baseline (*p* = 0.0003)
Rhee et al., (2013) [27]	South Korea	Mean: Supplement, 57.1 y; Placebo 58.5 y; AI; Postmenopausal	1. Supplement (n = 45) 5 mg of alendronate and 0.5-μg of active metabolite of vitamin D (Maxmarvil^®^) and 500 mg elementary calcium with 400 IU cholecalciferol (vitamin D) 2. Placebo (n = 46) placebo and 500 mg elementary calcium with 400 IU cholecalciferol (vitamin D) Duration: 24 weeks	**Bone mineral density** (lumbar, total hip, and femur neck) **Bone markers** (OCN, CTX, serum calcium, and phosphate)	↓**Lumbar bone mineral density** Placebo vs. supplement (*p* < 0.05) Placebo end vs. baseline (*p* < 0.05) (more pronounced in recently menopausal women) **Bone markers** ↑**CTX** Placebo vs. supplement (*p* < 0.05) Placebo end vs. baseline (*p* < 0.05) **↑OCN** Placebo vs. supplement (*p* = 0.08)
Shahvegharasl et al., (2020) [33]	Iran	Mean; Intervention, 44.9 y; Placebo 41.1 y; Stage I-III RBC; TAM; Premenopausal and postmenopausal	1. Vitamin D group (50,000 IU vitamin D3 weekly) (n = 22) 2. Placebo (n = 22) Duration: 8 weeks	**Body composition** (BMI, BW, WC, height, wrist) **Blood markers of inflam-mation** (hsCRP) **Angiogenic biomarkers** (Ang-2, VEGF-A, Hif-1)	**Angiogenic biomarkers** *premenopausal women* **↓Ang-2** Vitamin D end vs. baseline (*p* < 0.05) **↓VEGF-A** Vitamin D vs. placebo (*p* < 0.05) *Women with the absence of vascular invasion* **↓Ang-2** Vitamin D vs. placebo (*p* < 0.05) *Women with the infiltration of tumors into vascular or lymphatic vessels* **↑Hif-1** Vitamin D vs. placebo (*p* < 0.05)
Vani et al., (2016) [30]	India	Mean, 55.8 y; AI; Postmenopausal	1. Control (had adequate baseline serum vitamin D concentrations) no supplementation during study (n = 11) 2. Group 1 (had insufficient baseline serum vitamin D concentrations) (n = 60) supplementation with 2000 IU vitamin D3 and 1000 mg of calcium 3. Group 2 (had deficient serum vitamin D concentrations) (n = 11) supplementation with 4000 IU vitamin D3 and 1000 mg of calcium Duration: 12 weeks	**Body composition** (height, BW) **Bone markers** (Serum 25 (OH) vitamin D, phosphorus, calcium, PTH, alkaline phosphatase) **Quality of life** (HAQ-II)	**Bone markers** ↑**Serum 25 (OH) vitamin D** **↑Calcium,** **↑Phosphorus,** **↓PTH,** **↓Alkaline phosphatase activity** Group 1 + Group 2 end vs. baseline (*p* = 0.000) **↓Serum 25 (OH) vitamin D** Control group end vs. baseline (*p* < 0.05) Negative correlation between score (musculoskeletal symptoms) and serum 25 (OH) vitamin D concentration (*p* = 0.000)

**Abbreviations:** AIs: aromatase inhibitors; AIMSS: aromatase inhibitor-associated musculoskeletal symptoms; Ang: angiopoietin; BFI: brief fatigue inventory; BMI: body mass index; BPI-SF: brief pain inventory–short form; BRC: breast cancer; BW: body weight; CPIS: categorical pain intensity scale; CRP: C-reactive protein; CTX: serum C-telopeptide; FACT-B: functional assessment of cancer therapy breast; FACT-ES: functional assessment of cancer therapy–endocrine symptoms; HAQ II scores: health assessment questionnaire II; HCys: homocysteine; Hif: hypoxia-inducible factor; hs-CRP: high-sensitivity C-reactive protein; MENQOL: menopause-specific quality of life; MMA: methylmalonic acid; OCN: osteocalcin; TAM: tamoxifen; VEGF: vascular endothelial growth factor; WC: waist circumference; ↑: an increase or improvement in the associated value or measurement; and ↓: a decrease or deterioration in the associated value or measurement.

**Table 4:** Characteristics and results of studies with plant/fungi foods and supplements.

Author; Year	Country	Participants: Mean/Median Age; BRC Stage; Use of AI/TAM; Menopausal Status	Groups: n of Analyzed Samples; Duration of the Study	Evaluated Parameters	Significant Changes
Plant/Fungi Foods and Supplements					
Ávila-Gálvez et al., (2021) [36]	Spain	Mean; Intervention, 54 y; Placebo, 55 y; No neoadjuvant treatment; Some of them menopausal	1. Supplement (turmeric and red clover and flaxseed extracts and resveratrol; 296.4 mg phenolics per capsule) 3× day (n = 26) 2. Control (*n* = 13) Duration: 5 ± 2 days	**Disposition and metabolic profiles** (curcuminoids, isoflavones, resveratrol derivatives, and lignans)	**Phase-II metabolites** detected in urine, plasma, normal and malignant mammary tissue **Free curcumin** detected in normal and malignant mammary tissue in the supplement group
Braal et al., (2020) [39]	The Netherlands	Mean, 58.5 y; TAM	1. Green tea (1 g twice daily; containing 300 mg EGCG) and TAM (n = 7), 14 days 2. Control (TAM) (n = 7) 28 days Cross-over, n = 14 Duration: 42 days	**Pharmacokinetic interaction** between green tea supplements and endoxifen (active metabolite of TAM)	**Pharmacokinetic interaction** **Geometric mean endoxifen _AUC0–24h_** Green tea vs. control (*p* = 0.92) **C_max_** Green tea vs. control (*p* = 0.47) **C_through_** Green tea vs. control (*p* = 0.77)
Desideri et al., (2022) [40]	Italy	Mean, 59 y; Stage Ia-IIIb BRC; AI; Postmenopausal	1. Supplement (n = 46) α-Lipoic acid (240 mg) and Boswellia serrate (40 mg) and methylsulfonyl methane (200 mg) and Bromelain (20 mg)] OPERA^®^ Duration: 6 months	**Pain outcomes** AI-induced arthralgia (VAS, PRAI, CTCAE)	**Pain outcomes** **↓CTCAE scale** Supplement end vs. baseline (*p* = 0.0009) **↓VAS score** Supplement end vs. baseline (*p* = 0.0222). **↓PRAI score** Supplement end vs. baseline (*p* = 0.0001)
Ferraris et al., (2019) [37]	Italy	Mean; Supplement, 44.4 y; Placebo 44.6 y,; TAM/TAM and LHRH analogs; Premenopausal	1. Red clover (n = 42) (Promensil^®^ Forte, 80 mg red clover extract) and recommended Mediterranean diet 2. Placebo (n = 39) and recommended Mediterranean diet Duration: 24 months	**Body composition** (BMI, WC, HC) **Blood parameters** (Total cholesterol, HDL, LDL, TAG, glucose, HOMA-IR, estradiol, testosterone, DHEAS) **Cancer-related parameters** (endometrial thickness, breast density) **Quality of life** (menopausal symptoms)	**Body composition** **↓BMI** Red clover vs. placebo (*p* = 0.0013) Red clover end vs. baseline (*p* < 0.0001) **↓WC** Red clover vs. placebo (*p* = 0.04) Red clover end vs. baseline (*p* < 0.0001) **↓HC** Red clover end vs. baseline (*p* < 0.0001) **Blood parameters** **↑HDL** Red clover end vs. baseline (*p* = 0.01) Placebo end vs. baseline (*p* = 0.01) **Cancer-related parameters** **↓Breast density** Red clover end vs. baseline (*p* < 0.0001) Placebo end vs. baseline (*p* < 0.0001) ↓**Menopausal rating score** Red clover end vs. baseline (*p* < 0.0001) Placebo end vs. baseline (*p* < 0.0001) Red clover vs. placebo (*p* = 0.69)
MacGregor et al., (2005) [38]	United Kingdom	Median, 51 y; TAM/adjuvant chemo; With menopausal symptoms	1. Soy extract (235 mg with 17.5 mg of isoflavones) (n = 33) 2. Placebo (n = 35) Duration: 12 weeks	**Quality of life** (EORTC QLQ-C30, menopausal symptoms)	**Quality of life** **Menopausal symptoms** Soy vs. placebo (*p* > 0.05)
Radi et al., (2023) [35]	Iraq	Mean; Premenopausal 37.9 y; Postmenopausal 55.2 y; TAM/AI	1. Low-dose soy milk isoflavones (35 mg) (n = 120) 2. High-dose soy milk isoflavones (70 mg) (n = 120) Duration: 2 months	**Urinary estrogens levels** **and urinary isoflavones**	↓**Urinary level of estradiol** Low-dose soy milk isoflavones end vs. baseline (*p* < 0.0001) High-dose soy milk isoflavones (*p* < 0.0001) ↑**Urinary level of estrone** High-dose soy milk isoflavones (*p* < 0.0001) ↑**Urinary level of genistein** Low-dose soy milk isoflavones end vs. baseline (*p* < 0.0001) High-dose soy milk isoflavones (*p* < 0.0001) ↑**Urinary level of daidzein** Low-dose soy milk isoflavones end vs. baseline (*p* = 0.002) High-dose soy milk isoflavones (*p* < 0.0001) PostM vs. PreM Low-dose soy milk isoflavones (*p* = 0.039) PostM vs. PreM High-dose soy milk isoflavones (*p* = 0.03)
Simonavice et al., (2014) [34]	Georgia, USA	Mean, 64 y; Stage 0-III BRC; Finished/still on hormone suppressant therapies	1. Dried plum (90 g) and exercise (2 days/week of 10 strength and resistance training) (DP and E) (n = 11) 2. Exercise (E) strength training and resistance (n = 12) Duration: 6 months	**Body composition** (BMI, BW, BF, WC, HC, lean mass) **Muscular strength** (chest press, leg extension) **Bone markers** (BAP, TRAP-5b) **Blood markers of inflammation** (CRP)	**Muscular strength** ↑Upper and lower muscular strength DP + E end vs. baseline (*p* ≤ 0.05) E end vs. baseline (*p* ≤ 0.05) **Bone markers** ↓TRAP-5b DP + E end vs. baseline (*p* ≤ 0.05), E end vs. baseline (*p* ≤ 0.05)
Wen Su et al., (2024) [41]	Taiwan	Median, 57.4; Stage I-III BRC; TAM/AI	1. Supplement Ganoderma Microsporum immunomodulatory protein (n = 18) Once a day Duration: 6 months	**Quality of life** (QoL) **Blood markers of inflammation** (granulocytes, NK cells, NK cells with inhibitory surface markers, NK cells with activating surface markers, TNF-α, IL-6)	**Quality of life** ↑**Cognitive function** Supplement, 3 months vs. baseline (*p* < 0.05) Supplement, end vs. baseline (*p* > 0.05) **↓Fatigue** Supplement 3 months vs. baseline (*p* < 0.05) Supplement end vs. baseline (*p* > 0.05) **↓Insomnia** Supplement, end vs. baseline (*p* < 0.05) **Blood markers of inflammation** **↑CD19+ lymphocytes** **↓NKG2A+** **↓NKp30+ NK cells** Supplement, end vs. baseline (*p* < 0.05)

**Abbreviations:** AIs: aromatase inhibitors; BAP: bone-specific alkaline phosphatase; BF: body fat; BMI: body mass index; BRC: breast cancer; BW: body weight; CD19+: cluster of differentiation; CRP: C-reactive protein; DHEAS: dehydroepiandrosterone sulfate; HC: hip circumference; HDL: high-density lipoprotein; HOMA-IR: homeostatic model assessment for insulin resistance; LDL: low-density lipoprotein; NCI-CTCAE: National Cancer Institute–common toxicity criteria for adverse event; NK: natural killer; NKG2A+: natural killer group 2 member A; NKp30+: natural cytotoxicity receptor 3; PRAI: patient-reported arthralgia inventory; QoL: quality of life; TAG: triglycerides; TAM: tamoxifen; TRAP-5b: tartrate-resistant acid phosphatase isoform 5b; VAS: visual analog pain scale; WC: waist circumference; ↑: an increase or improvement in the associated value or measurement; and ↓: a decrease or deterioration in the associated value or measurement.

**Table 5:** Characteristics and results of studies with coenzyme Q_10_.

Author; Year	Country	Participants: Mean/Median Age; BRC Stage; Use of AI/TAM; Menopausal Status	Groups: n of Analyzed Samples; Duration of the Study	Evaluated Parameters	Significant Changes
Coenzyme Q10					
Premkumar et al., (2007) [43]	India	Median, 57 y; TAM	1. BRC, untreated (n = 84) 2. TAM and CoQ_10_ (100 mg) and riboflavin (10 mg) and niacin (50 mg) (n = 84) Duration: 90 days	**Blood markers of inflammation** (IL-1β, IL-6, IL-8, TNF-α, VEGF)	**Serum cytokine levels** **↓ IL-1β** **↓ IL-6** **↓ IL-8,** **↓ TNF-α** **↓ VEGF** TAM + CoQ_10_ end vs. baseline (*p* < 0.05) TAM + CoQ_10_ 45 days vs. baseline (*p* < 0.05) TAM + CoQ_10_ vs. BRC, untreated (*p* < 0.05)
Premkumar et al., (2008) [42]	India	Median, 57 y; TAM	1. BRC, untreated (n = 84) 2. TAM and CoQ10 (100 mg) and riboflavin (10 mg and niacin (50 mg) (n = 84) Duration: 90 days	**Pro-angiogenic markers**	↓**Levels of pro-angiogenic markers** TAM + CoQ_10_ end vs. baseline (*p* < 0.05) TAM + CoQ_10_ 45 days vs. baseline (*p* < 0.05) TAM + CoQ_10_ vs. BRC, untreated (*p* < 0.05)
Yuvaraj et al., (2009) [44]	India	Mean, 49 y; TAM; Postmenopausal	1. BRC, untreated (n = 78) 2. TAM and CoQ_10_ (100 mg) and riboflavin (10 mg) and niacin (50 mg) (n = 78) Duration: 90 days	**Blood parameters** (LPL, LCAT)	**Blood parameters** **↑Activity of LPL** TAM + CoQ_10_ end vs. baseline (*p* < 0.001) TAM + CoQ_10_ 45 days vs. baseline (*p* < 0.001)
Zahrooni et al., (2019) [45]	Iran	Mean, 40.7 y; Placebo, 36.3 y; Stage I-II BRC	1. CoQ_10_ 100 mg (n = 15) 2. Placebo (n = 15) Duration: 2 months	**Blood markers of inflammation** (IL-8, IL-6, VEGF)	**Blood markers of inflammation** **↓IL-8** CoQ10 vs. placebo (*p* < 0.05) **↓IL-6** CoQ10 vs. placebo (*p* < 0.05)

**Abbreviations:** AIs: aromatase inhibitors; BRC: breast cancer; IL-6: interleukin-6; IL-8: interleukin-8; LCAT: lecithin–cholesterol acyltransferase; LPL: lipoprotein lipase; TAM: tamoxifen; TNFα: tumor necrosis factor alpha; VEGF: vascular endothelial growth factor; ↑: an increase or improvement in the associated value or measurement; and ↓: a decrease or deterioration in the associated value or measurement.

## 4. Discussion

The objectives of this review were to apply a systematic approach and synthesize the available evidence on the impact of various components of food, the modified diet, and supplements on breast cancer health outcomes in patients undergoing adjuvant endocrine therapy with AIs or TAM. As is well established, all cancer survivors commonly experience a wide range of chronic health problems associated with both initial cancers and the consequences of cancer therapy. This is particularly applicable to breast cancer patients, as their survival rate is high, and life expectancy continues to rise [46]. However, the side effects of therapy and subsequent chronic health issues drastically impact patients’ physical, psychological, and social functions [47]. For example, and as addressed in the Introduction, the endocrine therapy that blocks the production of estrogen from the adipose tissue in postmenopausal women, despite being first-line treatment and less invasive compared to any chemo or radiation therapy, is not without side effects, especially considering that it is designed to last from 5 to 10 years. The most common side effects include vasomotor symptoms that manifest as hot flashes and/or night sweats, fatigue, musculoskeletal symptoms including arthritis-like pain, osteoporosis, and increased fracture risk, metabolic disturbances (including weight gain, diabetes, and dyslipidemia), vaginal atrophy, infertility in younger women, hair thinning, insomnia and other psychosomatic symptoms [48]. Unfortunately, these potential long-term complications are often underreported or tend to be ignored by health professionals. Additionally, many BRC patients are unaware of, inadequately prepared for, or unable to effectively manage their symptoms [49]. This could result in suboptimal adherence to therapy or early discontinuation of treatment and could ultimately negatively impact survival outcomes. Therefore, any dietary intervention (offering modifiable approaches) to alleviate these side effects needs to be investigated and critically evaluated, which is the main purpose of our study. Although Table 1, Table 2, Table 3, Table 4 and Table 5 depict all the studies, the discussion below is focused on the outcome parameters that were analyzed in two or more studies.

### 4.1. Body Composition and Weight as Outcomes

Long-term estrogen deprivation, such as that induced by antiestrogen therapy in BRC patients, significantly affects body composition, resulting in increased body weight and fat mass, and reduced muscle and bone mass, eventually leading to osteosarcopenic adiposity syndrome—the most detrimental stage in body composition deterioration [50,51]. The impairments in body composition may trigger or exacerbate other medical conditions, including hyperlipidemia, hypertension, diabetes, and cardiovascular disease, thus paying attention to them is of utmost importance.

Among the four nutritional intervention studies [20,22,34,37] evaluated in this review (Table 1, Table 3 and Table 4) that examined changes in body composition parameters with diet or additions to food, two incorporated concurrent exercise interventions to improve body composition [22,34]. For example, Papandreou et al. demonstrated that adherence to the Mediterranean diet with concurrent exercise decreased BMI, waist circumference (WC), body weight, and body fat in a group of women undergoing antiestrogen therapy [22]. That study demonstrated for the first time that a diet plan generated by the Clinical Decision Support System (CDSS) and creating personalized Mediterranean diet plans and physical activity guidelines could assist BRC patients, particularly during challenges like the COVID-19 pandemic. In another study [37], decreases in BMI and WC were significant in women following the Mediterranean diet and taking either red clover extract (80 mg of isoflavones daily) or placebo for 24 months. The benefits were more pronounced in the red clover group. The anti-obesity effect of red clover isoflavones, presumably formonectin, could be due to the inhibitory effects on α-glucosidase, preventing a rise in serum glucose [52] and thus inhibiting the signal for adipose tissue lipogenesis [53]. It is worth mentioning that apart from formonectin, other isoflavones of red clovers, such as genistein, daidzein, and biochanin A, have been shown to have estrogenic activity via binding to estrogen receptors [54]. As is well known, a lack of estrogen, such as in menopausal women, is associated with higher indices of obesity, while estrogen replacement therapy contributes to lowering visceral adipose tissue, as well as exerting benefits to bone and muscle [55,56].

However, such findings might not always align with the mainstay literature on weight loss, given that a decrease in weight/BMI leads to a decrease in estradiol and adiponectin levels [20]. Such effects were shown in a study of postmenopausal Mexican women (reviewed here, Table 1), who were in a group receiving a high-carbohydrate (68%), low-fat (12%) diet, compared to the control group receiving a standard diet with about 30% fat [20]. The decrease in adiponectin (secreted by adipocytes), otherwise important for energy homeostasis, was related to an overall decrease in adipose tissue resulting from the lower intake of fatty foods over six months.

In the study by Simonavice et al., interventions with dried plum (DP) (90 g/day) along with the resistant exercise training for BRCS on endocrine therapy did not show improvement in body composition, including bones, but were effective in maintaining it during the 6-month period [34] (Table 4). This was the first study to evaluate the combination of exercise and DP on both body composition and bone mineral density (BMD) in this population. Although DP did not enhance bone health in women with breast cancer as it did in healthy women [56], the study is significant as it highlights the positive impact of physical activity on bone health via a decrease in bone resorption, thus preventing unfavorable changes in body composition, which women could have experienced during the active disease [34]. The lack of positive results is probably due to the small sample size and lack of statistical power. Dried plums (prunes) are rich in phenolic compounds such as chlorogenic and neochlorogenic acids, exerting high antioxidative capacities. Despite the small sample size, this is a valuable study as it showed some additional beneficial outcomes of the dried plums/exercise intervention (discussed below). Further research is needed to explore the potential of some novel plant-based compounds, such as red clover extract and dried plum, and their ability to benefit body composition in BRCS.

It is well established that estrogen cessation in general, but particularly induced by estrogen deprivation therapy, alters bone turnover, reducing bone formation and increasing bone resorption, thereby increasing the risk of osteoporosis and subsequent fractures [57]. The results from the studies reviewed here [24,27] (Table 2 and Table 3) showed that supplementation with eicosapentaenoic acid (EPA), docosahexaenoic acid (DHA), and vitamin D can inhibit bone resorption in women undergoing AI treatment, suggesting that such supplementations may help reduce the risk of fractures in these patients. There was a noted decrease in bone resorption markers and serum C-terminal telopeptide (sCTX) levels in patients who were receiving 4 g of omega-3 (2520 mg EPA and 1680 mg DHA) daily for 3 months compared to those receiving placebo (safflower oil) [24]. Although both groups received calcium carbonate (1000 mg/day) and cholecalciferol (800 IU/day), whose effects on bones cannot be ruled out, there were indicators of the unique effects of omega-3 fatty acids, including the inverse association of serum EPA and DHA with sCTX. Both EPA and DHA can act as anti-inflammatory agents; however, there were no changes in markers of inflammation in this study. The authors suggest that the effects of the resolvins (final anti-inflammatory metabolites) derived from EPA and DHA might have reduced bone resorption and osteoclastogenesis [24]. The positive effects of omega-3 on bone have already been reported in postmenopausal women, and among the possible mechanisms, it was suggested that omega-3 fatty acids can improve circulation in the bone marrow and improve bone marrow cell health [58].

The most commonly used supplement among breast cancer patients is vitamin D, also frequently consumed in the general population for its numerous potential benefits. In line with this, the results from another study analyzed here [27] (Table 3) revealed that lumbar bone mineral density was decreased in the placebo, but not in the intervention group. Both groups received active metabolite of vitamin D (calcitriol) for 24 weeks, but the intervention group also received alendronate (an anti-bone resorption medication). There was no change in total hip and femur neck BMD in either group. However, serum bone turnover markers (sCTX and osteocalcin) were elevated in the placebo group compared to the intervention group [27]. These effects could be attributed to alendronate in the intervention group, although calcitriol might have diminished and slowed bone loss in the placebo group.

Overall, the studies on body composition demonstrated the diverse effects of dietary and supplement interventions in BRC patients undergoing endocrine therapy. Variations in intervention type, dosage, duration, and sample size likely influenced outcomes. Nonetheless, the evidence supports that, beyond a low-fat diet, other nutritional strategies, like bioactive food components (prunes, red clover) with their antioxidative and anti-inflammatory properties, along with exercise regimens, can positively affect body composition and survival in these patients. Additionally, these studies underscore the importance of bone health in this population, emphasizing that proactive measures can and should be taken, if not to enhance it then at least to preserve it. Unlike the other cancer patients with more advanced diseases causing anorexia, weight loss, cachexia, and other difficulties in eating, digesting, and absorbing nutrients [18,19], the BRC patients examined here were free of metastatic changes and free of chemo- and radiation therapy. They were also postmenopausal, thus suffering more from overweight/obesity and problems associated with excess adipose tissue. The interventions reviewed here enabled patients to normalize some of these conditions. Therefore, such interventional studies in other kinds of cancer patients should be of utmost priority in this research, as well as provide practical indications for the cancer team caring for the patient.

### 4.2. Cardiovascular Disease Risk Factors as Outcomes

The risk of cardiovascular disease (CVD) in women undergoing AI therapy is twofold. First is the treatment itself, as the reduction in estrogen increases the likelihood of cardiovascular events [59]. Second, women in menopause are already at an elevated risk of CVD due to hormonal changes [60]. It has been reported that endocrine adjuvant therapy in BRCS is associated with changes in important cardiovascular parameters, including total cholesterol and low-density lipoprotein cholesterol (LDL cholesterol) [61]. Several studies evaluated here (Table 1, Table 2 and Table 4) have examined the impact of various diets and supplements on lipid parameters and other CVD risk factors in this population [23,25,33,37,42,44].

For example, adherence to the Mediterranean diet together with physical activity maintained levels of triglycerides (TG), total cholesterol (TC), and LDL cholesterol and increased the level of high-density lipoprotein (HDL) cholesterol in women during hormonal therapy [22]. This diet provided higher levels of monounsaturated fatty acids, fiber, and vitamin C, which, along with exercise, improved blood glucose and lipid profiles and confirmed the beneficial effects of lifestyle modifications (including the addition of physical activity) in breast cancer management. On the other hand, supplementation with isoflavones from red clover [37], as well as with omega-3 fatty acids for 30 days [25] and 24 weeks [23], showed no significant changes in TC, HDL cholesterol, or LDL cholesterol.

However, combined supplementation with CoQ_10_, riboflavin, and niacin favorably altered the enzymes involved in lipid metabolism: lipoprotein lipase (LPL) and lecithin: cholesterol acyl transferase (LCAT) significantly decreased pro-angiogenic factors (Fibroblast Growth Factor, Hepatocyte Growth Factor, Epidermal Growth Factor Receptor, Transforming Growth Factor-beta, Thymidine Phosphorylase, and Prostaglandin E2) and increased anti-angiogenic marker levels (Endostatin and Trombospondin-1) in postmenopausal women during TAM therapy [42,44] (Table 5). Hypertriglyceridemia, hypovitaminosis, and cancer all contribute to a deficiency in LPL activity. Additionally, TAM treatment also influences lipid metabolism by decreasing LPL and LCAT activities. An increase in both enzyme activities in this study may have resulted from an interaction of CoQ_10_ with vitamin E, which regenerates it from its phenoxyl radical form enabling its action in both LPL and LCAT activity. Other studies also suggested that TAM treatment in BRC patients elevates apo A1 concentrations, which could further stimulate an increase in LCAT activity [62]. Additionally, CoQ_10_ may promote fatty acid oxidation through adenosine 5′ monophosphate-activated protein kinase (AMPK)-mediated stimulation of the peroxisome proliferator-activated receptor α (PPARα), which in turn increases the expression of LPL and apolipoprotein A1 (apo A1), potentially reducing TG and very-low-density lipoprotein levels in patients with diabetes type 2 [63]. This suggests the potential benefits of such supplements and interventions for the cardiovascular health of BRCS undergoing TAM.

Additionally, supplementation with 50,000 IU/week of cholecalciferol for 8 weeks reduced the serum levels of angiogenic biomarkers like vascular endothelial growth factor (VEGF)-A, angiopoietin (Ang)-2, and hypoxia-inducible factor (Hif)-1 in BRCS. The beneficial effects varied by tumor invasiveness into the vascular tissue [33]. In patients without vascular invasion (the presence of tumor cells within the lumen of blood and/or lymph vessels), cholecalciferol significantly lowered the Ang-2 levels. At the same time, cholecalciferol significantly increased Hif-1 in those with vascular or lymphatic invasion (the presence of tumor cells within the lumen of blood and/or lymph vessels). These findings suggest that cholecalciferol can decrease angiogenic biomarkers in BRCS depending on tumor invasiveness into vessels, but further studies with larger cohorts are needed to confirm these effects [33]. In contrast, a high dose of oral cholecalciferol supplementation in non-diabetic patients with chronic kidney disease stages 3–4 showed insignificantly reduced levels of Ang-2 and no changes in other angiogenic markers, including Ang-1, vascular endothelial growth factor receptor (VEGFR), VEGF, and tyrosine kinase receptor-2 after 16 weeks [64]. The dissimilar results between the studies suggest that the effects of supplementation may depend on several factors, including the duration of the intervention and the specific characteristics of the disease.

Taken together, these findings highlight the importance of analyzing dietary and supplement interventions as a means of mitigating CVD risk in women with breast cancer history undergoing endocrine therapy, and further examining products and foods that can contribute to their cardiovascular health improvement. This is particularly important for this segment of population (overweight/obese, pre- to postmenopausal women), which is also at the highest risk of various cardiovascular diseases. Despite the fact that this issue is important, there is a lack of randomized clinical trials with more specific CVD conditions, as well as with various types of cancers. This provides an excellent opportunity to expand research in patients with cancer and coexisting CVD and give a head-start to health professionals in dealing with possible more serious complications.

### 4.3. Inflammation as an Outcome

Cancer and its treatments are closely linked to elevated inflammatory processes [65]. Numerous inflammatory factors, including interleukins, tumor necrosis factor alpha (TNF-α), interferon, various chemokines, transcription factors, and lipid metabolites such as leukotrienes, prostaglandins, thromboxanes, and pro-resolving molecules, play pivotal roles in regulating the initiation, progression, and resolution of inflammation [65]. C-reactive protein (CRP) is released in response to pro-inflammatory cytokines like interleukin-6 (IL-6), and is often elevated in cancer patients, thus serving as a marker of inflammation [66]. The impact of some dietary supplementations on these inflammatory markers was observed in several studies evaluated in this review [23,24,25,26,34,41,43,45] (Table 2, Table 3, Table 4 and Table 5).

A reduction in mean serum CRP concentration (−2.8 mg/L) was noted after 30 days of treatment with EPA/DHA/hydroxytyrosol/curcumin capsules [25]. Conversely, supplementation with omega-3 fatty acids alone for 3 months resulted in an increase in CRP levels in women on AI therapy [24], or had no significant impact on CRP after 24 weeks of supplementation [23]. Hutchins-Wiese et al. [24] speculated that the increase in CRP could be attributed to acute phase response; however, further research is necessary to clarify this effect. One of the differences between the studies with no effect and those showing beneficial impacts on CRP is that the latter were conducted with the addition of hydroxytyrosol/curcumin [25]. The in silico approaches (studies performed via computer simulation) also reported that both hydroxytyrosol [67] and curcumin [68] can directly interact with CRP. Additionally, a recent meta-analysis reported no effect of omega-3 supplementation on CRP in hospitalized cancer and other patients [69]. Similarly, vitamin D supplementation for 8 weeks also did not show a decrease in CRP [33]. The reasons for this are beyond the scope of this review; however, the authors point out the small sample size and the fact that most of the women were in an advanced stage of BRC.

In the study with DP consumption (discussed above with regard to bone and body composition) with and without resistance exercise, there was no significant influence on CRP levels, but both groups moved from high- to moderate-risk categories (CRP level decreased from 3 mg/L to 1–3 mg/L) of cardiovascular disease, which is clinically significant due to CRP’s association with CVD risks and mortality [34].

In two studies applying intervention with CoQ_10_, there was a serum IL-6 reduction after 2 months [45] and also after 3 months [43] in BRCS receiving TAM therapy. This is in line with the conclusions from the recent meta-analysis of randomized clinical trials reporting that CoQ_10_ reduced levels of IL-6 in the general population [70]. This effect could be explained by various mechanisms, such as the downregulation of nuclear factor kappa B or the activation of PPAR-mediated anti-inflammatory responses [70].

The results from the Lustberg et al. [26] study showed that supplementation with a high dose of EPA and DHA for 12 and 24 weeks had minimal effect on the serum IL-6 levels in BRC patients undergoing AI therapy. The authors pointed out the lack of a control healthy group for comparison and suggested the possible inhibitory effects of AIs on omega-3 effects [26].

The results of the Taiwanese pilot study conducted over 6 months demonstrated an improvement in circulating immune cell composition following supplementation with the medicinal fungi *Ganoderma Microsporum* immunomodulatory protein, suggesting potential benefits in modulating immune responses [41]. Possible mechanisms (shown in vitro) include the modulation of the nuclear factor E-2 related factor 2 (Nrf2) signal pathways. Both Nrf1 and Nrf2 mitigate oxidative stress, enhance cellular resilience, and induce new mitochondrial synthesis [71].

The variation in the type of therapy received by patients (AI and/or TAM therapy), differences in the anti-inflammatory properties of the supplements and foods used, and baseline differences in inflammatory status among participants, are all factors that potentially influenced the observed outcomes. Further research, including larger and longer clinical trials with higher but still safe doses, is needed to validate and generalize these findings and explore the broader impact of these interventions on inflammation and immune function in cancer patients undergoing endocrine therapy. Such research should not be limited to just breast cancer patients, but should include other more difficult cases and those with metastatic developments, in light of the fact that not only cancer cells and their invasions of other tissues, but various cancer therapies, promote and uphold a constant pro-inflammatory state.

### 4.4. Quality of Life as an Outcome

The majority of studies reviewed here examined the impact of nutritional interventions on changes to the quality of life (QoL) of BRC patients. The European Organization for Research and Treatment of Cancer (EORTC)’s Quality of Life Questionnaire (EORTC QLQ-C30) and EORTC questionnaire for assessing the quality of life in breast cancer patients (EORTC QLQ-BR23) were the most frequently used tools for assessing QoL in breast cancer survivors [72]. Other commonly used questionnaires included the Functional Assessment of Cancer Therapy–General (FACT-ES) questionnaire addressing the endocrine symptoms related to cancer treatment; the Brief Pain Intensity Score (BPI-SF) questionnaire addressing the pain severity and its impact on functioning; and the general survey for all, known as the 36-Item Short-Form Survey (SF-36). Additionally, the Cancer Fatigue Scale (CFS) was often used to assess the severity of fatigue, a common symptom in cancer patients. Although fatigue is also prevalent in the general population and linked to a stressful lifestyle, it is notably more intense and persistent in cancer patients throughout all phases of cancer treatment [73]. A poor QoL in cancer survivors may lead to the termination of therapy, and thus an increased risk of cancer recurrence and mortality. Interventions such as exercise, diet, and lifestyle changes can help manage fatigue and improve QoL, as evaluated here (Table 1, Table 2, Table 3 and Table 4) and discussed below.

In a study conducted by Papandreou et al., the Mediterranean diet and physical activity improved the quality of life for BRC patients during the COVID-19 pandemic through positive effects on global health, role functioning, and emotional functioning, as assessed by the EORTC-QLQ-C30 questionnaire (Table 1) [22]. Similarly, a diet rich in vegetables and fruits, in combination with resistance and aerobic exercise, improved some aspects of physical functioning and vitality in overweight/obese patients while undergoing endocrine therapy [21]. However, neither diet only nor exercise only showed improvement compared to the control group. It needs to be noted that this was a complex study, as it included various interventions, either with food or exercise only, and lasted up to 52 weeks. The findings suggest that combining exercise and diet may be the optimal lifestyle intervention for improving QoL, particularly in overweight or obese BRC survivors.

Results from the study on magnesium supplementation for 4 weeks also demonstrated reductions in fatigue, as well as in abnormal sweating, hot flashes, and distress, all associated with menopausal symptoms [28]. The precise mechanism by which magnesium reduces hot flashes remains unclear. However, magnesium is known to exhibit neuroprotective and vaso-protective properties, and may contribute to increased serotonin levels in the brain [28]. Hot flashes are associated with imbalances in serotonin and norepinephrine. An increase in serotonin levels has been shown to improve these symptoms [74]. The evidence also suggests that magnesium deficiency is linked to depression, and that supplements may enhance the efficacy of antidepressant medications, which also elevate serotonin levels, thereby further improving symptoms in depressive patients [75].

Numerous studies suggest that soy isoflavones exhibit multiple health benefits (similar to estradiol). These include reducing cholesterol levels and providing cardioprotective benefits [76], enhancing bone health [77], reducing hot flash frequency and severity [78], improving menopausal symptoms in both perimenopausal and postmenopausal women [79], and lowering the risk of lung, prostate [80], and breast cancers [81]. Nevertheless, the results from the study investigating the supplementation of 70 mg of isoflavones daily from soy extract, in soy capsule form, showed no significant effects on menopausal symptoms after 12 weeks [38]. This study was conducted in relatively young women (median 51 years) with early onset of breast cancer, which might have concealed the outcomes.

Among the various side effects associated with AI therapy, joint pain and stiffness are the most frequent, generally named AI-induced arthralgia (AIA) [46]. Supplementation with omega-3 fatty acids commonly used by BRC patients [82,83], as well as vitamin D and other supplements, shows varying effects on AIA. As evaluated here, Martínez et al. (2019) (Table 2) found that supplementation with EPA, DHA, hydroxytyrosol, and curcumin (460 mg and 12.5 mg and 47.5 mg/day, respectively) reduced pain by 21.5% during AI therapy [25]. However, Lustberg et al. [26] reported that supplementation with a high dose of omega-3 fatty acids (4.3 g/day) showed no significant influences on pain severity after 12 and 24 weeks of intervention. Similar results were also reported earlier [84]. Furthermore, Lustberg et al.’s [26] group reported that quality of life, assessed by FACT-ES scores, decreased significantly in the placebo group and remained unchanged in the omega-3 group at 12 weeks, but not at 24 weeks [26]. Similarly, in a study conducted by Hershman et al., (2015), 560 mg EPA and DHA did not significantly improve arthralgia at weeks 6, 12, and 24 in BRC patients on AIs [23]. However, Shen et al. (2018) found that this supplementation was associated with a significant reduction in AIA in obese breast cancer patients compared to a placebo [85]. Overall, while omega-3 fatty acid supplementation may offer modest relief from the side effects of AI therapy, its effectiveness may vary based on other unique factors, including obesity. The potential short-term benefits of high doses of (EPA and DHA) early in AI treatment might have a limited impact as side effect progress [26].

Other studies presented here also highlight the benefits of supplementation for pain management. For example, Campbell et al. (2018) reported a 34% average improvement in pain, as assessed by the BPI-SF questionnaire, following 90 days of daily supplementation with 2.5 g of vitamin B_12_. Furthermore, analysis of FACT-ES scores demonstrated improvements in all scales, indicating that vitamin B_12_ could be a safe and effective option for mitigating side effects associated with AI treatment [31]. Such results are reasonable to expect, as it is well known that B_12_ has a crucial role in muscular regeneration and nervous system functioning (through myelin sheet repair), as well as in red blood cells and DNA synthesis.

Similar benefits, but from studying different supplements, were shown in other studies. For example, Desideri et al. (2022) found that a combination of alpha-lipoic acid, Boswellia serrata, methylsulfonylmethane, and bromelain led to significant reductions in arthralgia-related pain after 24 weeks of use [40]. In this context, alpha-lipoic acid neutralizes reactive oxygen species, inhibits their generation, and improves the functioning of other key antioxidants such as vitamin E, vitamin C, and glutathione. These interactions mitigate oxidative stress and may indirectly contribute to pain reduction [86]. Boswellic acids exert anti-inflammatory effects by inhibiting enzymes like 5-lipoxygenase. Methylsulfonylmethane has chondroprotective properties through promoting cartilage synthesis, while bromelain, as a proteolytic enzyme, reduces edema [40]. Additionally, Desideri et al. (2017) also reported that this supplement improves chemotherapy-induced peripheral neuropathy in patients previously treated with neurotoxic chemotherapy agents [87]. However, given the variation in results, additional randomized, double-blind studies are needed to further confirm the effectiveness of these dietary supplements in patients undergoing endocrine therapy.

Regarding vitamin D, there is some evidence supporting high-dose vitamin D as a potential therapeutic option, but the overall findings remain mixed. For instance, Vani et al. (2016) (Table 1) reported that 12 weeks of vitamin D3 and calcium supplementation led to a reduction in arthralgia symptoms in postmenopausal, estrogen receptor-positive breast cancer patients receiving letrozole for more than two months [30]. Based on these findings, they recommended adjusting the supplementation dose according to serum 25(OH) vitamin D levels at 6 and 12 weeks, to optimize vitamin D status and alleviate musculoskeletal symptoms. Also, vitamin D (30,000 IU/week) for 24 weeks significantly prevented joint pain from worsening in women also receiving letrosol [29] (Table 1). However, in a study by Niravath et al. (2019) (Table 1), neither high nor standard doses of vitamin D and calcium over 52 weeks were found to prevent the development of AIA or diminish joint/muscle pain [32]. The authors emphasize that vitamin D likely does not play a significant role in AIA for the majority of patients, and that the reasons for discrepancies between studies remain unclear. However, a host of factors, most importantly, individual patient characteristics, may contribute to the variability in responses to vitamin D. Given the mixed results of existing trials examining vitamin D as a treatment for AI-induced arthralgia, further research is needed to determine the optimal dosing and target levels of vitamin D. Continued research is essential to better understand the etiology and treatment of AIA, as it remains a significant cause of non-compliance with AI therapy in breast cancer survivors.

In summary, improving QoL in breast cancer survivors involves a holistic approach and the integration of dietary and physical activity interventions alongside targeted nutritional supplementation. These interventions can alleviate fatigue, enhance physical functioning, and improve overall vitality, ultimately contributing to better QoL and potentially reducing the risk of cancer recurrence and mortality. A particularly thorough and useful analysis of various dietary recommendations to alleviate many symptoms in various cancers and improve the QoL of patients was published recently [18]. Briefly, the authors recommended the initial assessment of the patient’s dietary intake and subsequent regular follow-up, which is a primary task of any dietitian/nutritionist, but is not always followed-up or recognized by other health professionals in the team [88]. Furthermore, Garutti et al. [18] provided an overview of the most prevalent and painful conditions that cancer patients have to deal with, including anorexia and cachexia, diarrhea, nausea and vomiting, stomatitis, and xerostomia, to name just a few. They also proposed some referent guidelines and roadmaps for treatment with optimal nutritional strategies and even included customized recipes for specific situations of cancer patients.

### 4.5. Additional Possibilities with Diet Interventions

#### 4.5.1. Cyclic Fasting-Mimicking Diets and Their Role in Breast Cancer

In addition to the original studies on the hormone-receptor-positive BRC patients receiving endocrine therapy reviewed and presented here, we want to provide a brief insight into and discuss the most recent and relevant research in this area. Although some of these studies were conducted on cell cultures or animals or they report the preclinical trials’ results (all exclusions for our review), they warrant inclusion in this discussion due to their novel and meritorious content.

In this context, the most interesting approaches to mitigating cancer recurrence and enhancing the effects of endocrine therapy could be the implementations of the intermittent fasting and/or fasting-mimicking diets [89,90]. Even earlier, periodic energy-restricted diets (without malnutrition) were shown to exert multiple health benefits, including metabolic and anti-inflammatory benefits as well as extend life expectancy [91]. However, research on the beneficial role of intermittent fasting in breast and other cancer treatment is just emerging. For example, in the mouse models of hormone-receptor-positive breast cancer, periodic fasting or a fasting-mimicking diet (FMD) enhanced the activity of tamoxifen and fulvestrant by lowering circulating IGF-1 as well as insulin and leptin concentrations. The treatment with fulvestrant combined with cyclic FMD showed tumor regression and lower resistance to drug treatment [89]. Similarly, the recent review of human preclinical and clinical (1- and 2-phase) trials by Vernieri et al. [90] revealed that cyclic fasting and an FMD produced synergistic antitumor effects when combined with standard-of-care treatments, and protected normal tissues from adverse effects. Based on the “International Consensus for Fasting Terminology”, the FMD in humans typically refers to a diet low in carbohydrates and protein and relatively high in fat, to meet an overall energy intake of about 1000 kcal. Such a diet should be adhered to for 3–4 days every 3–4 weeks [92]. This diet and similar intermittent fasting diets are designed bearing in mind the hallmarks of cancer cells’ ability to reprogram and assume unrestrained proliferation, invasion of the surrounding tissues or distant organs, and evade/neutralize the defense activity of the immune system [93]. Therefore, the reduction in overall energy intake, but particularly in carbohydrates, protein, and to some extent fat, results in lower concentrations of serum glucose, amino acids, and certain growth factors (insulin, leptin, and IGF-1), as well as decreased fatty acid synthesis in the host, leading to the inhibition of cancer cell growth and presumably also to the synergistic action with anticancer medications [90]. Therefore, especially in hormone-receptor-positive breast cancers, the FMD inhibits some of the enzymes and molecules, such as the mitogen-activated protein kinase (MAPK), PI3K-AKT, mechanistic target of rapamycin (mTOR), and other crucial metabolic pathways, involved in glucose uptake, glycolysis, and the tricarboxylic acid cycle, as well as the coupling of oncogenic signaling with metabolic reprogramming. Therefore, all of these pathways providing cancer cells with the energy and metabolites necessary to survive, grow, proliferate, and metastasize are inhibited [93,94].

The beneficial effects of energy and some nutrient restriction in various cancers have been previously studied, along with suggestions for some possible mechanisms. In fact, the 1931 Nobel Prize was awarded to Otto Warburg for his discovery of the fact that tumor cells are highly dependent on the availability of glucose (known as the “Warburg effect”) [95]. Subsequently, it was reported that other dietary components, such as individual amino acids, fatty acids, and/or other nutrient metabolites, can modulate the proliferation of different cancer cells, even within the same tumor. Sometimes, these restrictions have not been potent enough to inhibit cancer cell growth and/or their metastatic expansion. Therefore, it became difficult to determine which combination and amounts of specific nutritional/metabolic restrictions can lead to tumor regression or prevent resistance to these nutrients in cancer treatments. Additionally, while the FMD and other cyclic fasting procedures are relatively easy to implement in cell cultures or animal models, this is not the case when cancer patients are involved, especially not for a longer time during therapy, creating another limitation. Therefore, the need for more feasibility studies and/or randomized clinical trials in this area is of utmost urgency and necessity. Only such studies will be able to provide evidence on the effects of the combination of the FMD with some standard-of-care therapies and whether it can reduce tumor growth, increase patients’ survival, or enhance their quality of life.

#### 4.5.2. Whole Grains and Their Role in Breast Cancer

Another unexplored yet promising dietary regimen for breast cancer prevention and/or its recurrence could be a high intake of whole-grain cereals, in view of quite impressive evidence on their antioxidant, anticancer, and anti-inflammatory effects, supported by epidemiological and clinical studies, as reviewed earlier [19]. As is currently understood, the overall health benefits of whole grains in cancer patients could be due, not only to their high fiber content and the fact that they provide satiety and the possibility of modifying weight and body composition, but also to their presence of various nutraceuticals and bioactive components [96]. These include anthocyanins (like lignans, flavones, and tannins); carotenoids pigments (like α-carotene/β-carotene); phytosterols (like sterols and stanols); non-starchy polysaccharides (like insoluble dietary fiber and β-Glucans); vitamin E metabolites; and tocols (like tocopherols and tocotrienols). All of these are components of whole wheat, rice, barley, rye, oat, millet, and sorghum, representing a staple diet in many cultures and the main source of energy. Therefore, in addition to being rich in bioactive components and presenting as safe and natural foods, whole-grain cereals present an agreeable and relatively cheap choice for widespread, long-term use, not only in breast cancer patients but in other diverse populations. Their mechanism of action is attributed to the ability of these phytochemicals to act on a molecular basis and influence cancer cell proliferation by causing cell death, to affect pro-inflammatory cytokines and tumor-specific T cells, and also to inhibit angiogenesis and breast cancer stem cell division, as well as to positively regulate the entire tumor microenvironment [19].

However, there are several limitations in the studies reporting the use of whole-grain cereals in cancer patients. For example, in some epidemiological studies it is unclear which grains were used, and even more so which specific phytochemicals were investigated to assess their benefits, and on which type of breast cancer cell. The content of phytochemicals is different in different species of grains as well as in the different geographical region where each one is grown. Xie et al. [19] excellently reviewed this whole issue.

Overall, all these results reinforce the need for further clinical studies of cyclic fasting-mimicking and whole grain diets, as an adjuvant to estrogen therapy in hormone-receptor-positive breast cancer patients, as well as investigation of other dietary approaches including additions to foods and supplements, as reviewed here and also elegantly tackled by Garuttri et al. and Xie et al. [18,19].

### 4.6. Limitations

This review presents a comprehensive evaluation of the current literature regarding the effects of foods and supplements on anthropometric and biochemical parameters, bone health, inflammation markers, and quality of life among breast cancer survivors. Many studies have been evaluated, each with certain limitations that have been to some extent reflected in this review. It is important to note that the studies varied in duration, and that there were discrepancies in the use of TAM or AIs before baseline data collection in some studies. Additionally, some of the studies were conducted without a control group, and/or involved a limited number of participants. Furthermore, ethnicity or race was not reported in most of the studies. Consequently, the results should be interpreted with caution.

The heterogeneity in the types of supplements used (animal, herbal, fungal, vitamins, and minerals) as well as doses of the same supplements (omega-3 fatty acids from 460 mg to 4.3 g), different intervention durations (from 5 days to 24 months), the varied sample sizes (from 13 to 107 participants), the different durations that women were on endocrine therapy, and differences in menopausal status among participants are all reasons contributing to variability in the findings of the evaluated studies. Each of these was mentioned in the Discussion section under separate titles (Body composition, CVD risks, Inflammation, and QoL) and they are also listed in the respective Table 1, Table 2, Table 3, Table 4 and Table 5. Additionally, we also discussed some of the newest treatments (cyclic fasting-mimicking diets and whole-grain cereal diets) in breast cancer. Unfortunately, due to their novelty and the limited research in clinical trials, the results of these studies were off limits and were not included in the tables or evaluated together with interventional, randomized clinical trials.

## 5. Conclusions

This review examined the effects of the different types of nutritional intervention and physical activity (just three studies) in breast cancer survivors during endocrine therapy. Different outcome measures were evaluated as well. In general, the evidence suggests that adherence to dietary patterns, such as the Mediterranean diet, a low-fat diet, and a high intake of fruit and vegetables, was beneficial for several outcomes, in addition to physical activity, although only three studies incorporated it. Specifically, supplementation with some foods (dried plum and red clover extract), adherence to the Mediterranean diet, and a low-fat diet contributed to improving or maintaining body composition, including body weight, WC, and bone health, especially in overweight or obese patients. Furthermore, supplementation with vitamin D or omega-3 fatty acids improved lipid and angiogenic parameters and QoL. In some instances, these supplementations were not effective, but they also did not exert any negative consequences, even when added in considerable amounts. While all these results are promising, it is hard to summarize the outcomes of each supplement, reach a unified conclusion for each, or possibly propose a universal treatment. This is due to the diverse nature of the study designs, patients, and supplement dosages, as elaborated upon throughout the Discussion section and summarized in the Limitations section. What is important to note is that the research into both the identification of the molecular and genetic classifications of breast cancer cell types and subsequent nutritional and other lifestyle modifications is growing, to counter and synergistically operate with standard-of-care treatments, making this a promising field for patients and their healthcare teams. Nevertheless, further well-designed research with a large sample size is needed to explore the effects of specific nutritional interventions and dietary adherence on body composition, metabolic parameters, and QoL in BRC survivors during endocrine therapy.

### Closing the Gaps and Recommendations for Future Research

Based on the findings of our review on breast cancer patients, we identified some gaps or less-investigated foods/nutrients in the current literature. Thus, we propose the following specific points for the advancement of the field:

1. Long-term studies on the combined effects of dietary patterns and pharmacological treatments on body composition, cardiovascular diseases, inflammation, and QoL, as well as the other numerous consequences of cancer treatment.
  (a) Regarding body composition, beyond a low-fat diet and reduced energy intake, other nutritional strategies, like bioactive food components with their antioxidative and anti-inflammatory properties (see more under item #3), along with exercise regimens and lifestyle modifications, can positively affect body composition and survival in these patients. Additionally, these studies underscore the importance of bone health, not addressed unless in metastatic bone cancer patients;
  (b) CVD risk is particularly important for this segment of the population (overweight/obese, pre- to postmenopausal women). Despite the importance of the coexistence of both conditions, there is a lack of randomized clinical trials with more specific CVD conditions, as well as with various types of cancers;
  (c) Chronic inflammation is a hallmark of any cancer and subsequent chemo- or radiation therapies. Therefore, the research should not be limited to just breast cancer patients, but should include other more difficult cases and those with metastatic developments;
  (d) The worsening of quality of life is an important side effect of any cancer and its treatment, affecting both the physiological and psychological well-being of patients. Therefore, improving QoL in breast cancer survivors must involve a holistic approach and the integration of dietary and lifestyle interventions to alleviate the many side effects and enhance physical functioning, ultimately contributing to faster recovery and reducing the risk of cancer recurrence and mortality.
2. Exploration of cyclic fasting-mimicking diet regimens in diverse cancer patients, particularly in those with metastatic changes, and their synergism with standard-of-care treatment or other pharmacological agents. Since this research is in its infancy, more animal studies and phase-2 clinical trials will be needed to progress to randomized, phase-3 clinical trials. However, it is a very promising and novel approach.
3. Evaluation of whole-grain cereal composition and its potential as a preventative strategy for breast cancer initiation or its recurrence is also an unexplored topic with great potential. As addressed above, these foods are rich in fiber and bioactive components and present a safe, natural, and relatively cheap choice for widespread, long-term use, not only in breast cancer patients but in other diverse populations. Unfortunately, due to the limited studies in cancer patients and the limitations of the existing studies, it is still unclear which grains were used (from which geographic region) and which specific phytochemicals showed beneficial effects on specific types of cancer cells.

All these findings could serve as suggestions for guiding policy initiatives and nutritional and lifestyle modifications for cancer prevention and possible synergistic treatment with standard-of-care therapies, to alleviate side effects and diminish cancer recurrence.

## Abbreviations

The following abbreviations are used in this manuscript:

AIs	Aromatase inhibitors
AIA	Aromatase inhibitor-induced arthralgia
AIMSS	Aromatase inhibitor-associated musculoskeletal symptoms
AMPK	Adenosine 5′ monophosphate-activated protein kinase
Ang	Angiopoietin
apo A1	Apolipoprotein A1
BAP	Bone specific alkaline phosphatase
BFI	Brief fatigue inventory
BFM	Body fat mass
BMD	Bone mineral density
BMI	Body mass index
BPI-S	Brief Pain Intensity Score
BPI-SF	Brief pain inventory–short form
BRC	Breast cancer
BRCS	Breast cancer survivors
BW	Body weight
CD19+	Cluster of differentiation
CDSS	Clinical Decision Support System
CFS	Cancer Fatigue Scale
CoQ_10_	Coenzyme Q_10_
CPIS	Categorical pain intensity scale
CRP	C-reactive protein
CTX	Serum C-telopeptide
CVD	Cardiovascular disease
DHA	Docosahexaenoic acid
DHEAS	Dehydroepiandrosterone sulfate
DPD	Deoxypyridinoline
DP	Dried plum
EORTC	The European Organization for Research and Treatment of Cancer
EORTC QLQ-BR23	European organization for research and treatment of cancer quality of life questionnaire the breast cancer supplementary module
EORTC QLQ-C30	European organization for research and treatment of cancer quality of life questionnaire core 30
EPA	Eicosapentaenoic acid
ER	Estrogen receptor
FACT-B	Functional assessment of cancer therapy breast
FACT-ES	Functional Assessment of Cancer Therapy–General
FMD	Fasting-mimicking diet
HADS	Hospital anxiety and depression scale
HAQ II scores	Health assessment questionnaire II
HC	Hip circumference
HCys	Homocysteine
HDL	High-density lipoprotein
Hif	Hypoxia-inducible factor
HOMA-IR	Homeostatic model assessment for insulin resistance
HR+	Hormone-receptor-positive
hs-CRP (CRP)	High-sensitivity C-reactive protein
IFNγ	Interferone gamma
IGF-1	Insulin-like growth factor 1
IL-1	Interleukin-1
IL-6	Interleukin-6
IL-8	Interleukin-8
IL-10	Interleukin-10
IL-15	Interleukin-15
IL-17	Interleukin-17
LC	Long chain
LCAT	Lecithin: cholesterol acyl transferase
LDL	Low-density lipoprotein
LDL cholesterol	Low-density lipoprotein cholesterol
LPL	Lipoprotein lipase
MAPK	Mitogen-activated protein kinase
MDA	Malondialdehyde
MedDiet score	Mediterranean diet score
MENQOL	Menopause-specific quality of life
MMA	Methylmalonic acid
mTOR	Mechanistic (mammalian) target of rapamycin
NCI-CTCAE	National Cancer Institute–common toxicity criteria for adverse event
NK	Natural killer
NKG2A+	Natural killer group 2 member A
NKp30+	Natural cytotoxicity receptor 3
Nrf2	Nuclear factor E-2 related factor 2
NS	Not significant
OCN	Osteocalcin
P1NP	Procollagen type 1 N-terminal propeptide
PPARα	Peroxisome proliferator-activated receptor α
PRAI	Patient-reported arthralgia inventory
PTH	Parathyroid hormone
QoL	Quality of life
SAA	Serum amyloid A
sCTX	Serum C-terminal telopeptide
SF-36	36-Item Short-Form Survey
TG	Triglycerides
TAM	Tamoxifen
TC	Total cholesterol
TG	Triglycerides
TGFβ	Transforming growth factor beta
TNFR-2	Tumor necrosis factor receptor 2
TNF-α	Tumor necrosis factor alpha
TRAP-5b	Tartrate-resistant acid phosphatase isoform 5b
VAS	Visual analog pain scale
VEGF	Vascular endothelial growth factor
VEGFR	Vascular endothelial growth factor receptor
WC	Waist circumference
